# The Identification of Boll Weevil, *Anthonomus grandis grandis* (Coleoptera: Curculionidae), Genes Involved in Pheromone Production and Pheromone Biosynthesis

**DOI:** 10.3390/insects12100893

**Published:** 2021-10-01

**Authors:** Lindsey C. Perkin, Jose L. Perez, Charles P.-C. Suh

**Affiliations:** Insect and Cotton Disease Research Unit, USDA Agricultural Research Service, College Station, TX 77845, USA; jose.perez@usda.gov (J.L.P.); charles.suh@usda.gov (C.P.-C.S.)

**Keywords:** boll weevil, pheromone production, RNA-seq, transcriptome

## Abstract

**Simple Summary:**

The boll weevil is a destructive pest of commercial cotton throughout the Americas. An eradication program in the United States has removed the boll weevil from most of its range. However, weevil populations in South Texas remain a threat to eradicated areas. Pheromone traps are used to monitor boll weevil activity, and when a weevil is captured, eradication programs rely on malathion for control. However, the effectiveness of pheromone traps in detecting incipient boll weevil populations is reduced during certain times of the year. Additionally, human safety and environmental concerns, as well as the potential development of malathion-resistant populations, have prompted program managers to seek alternative control methods. We sequenced and compared pheromone-producing and non-pheromone-producing weevils to identify genes involved in pheromone production, which, in turn, could be an environmentally friendly way to target gene-level pest control that is specific to the boll weevil. Our results revealed genes involved in pheromone production, as well as insect development and immunity, which may be targeted for boll weevil suppression.

**Abstract:**

Eradication programs for the boll weevil, *Anthonomus grandis grandis* Boheman (Coleoptera: Curculionidae), rely almost exclusively on pheromone traps to indicate the need for insecticide applications. However, the effectiveness of traps in detecting weevil populations is reduced during certain times of the year, particularly when cotton is actively fruiting. Consequently, this could result in fields becoming heavily infested with weevils. It is widely speculated that the lack of weevil captures in traps during this period is largely due to the overwhelming amount of pheromone released by weevils in the field, which outcompete the pheromone released from traps. Thus, this work sought to identify genes involved in pheromone production so that new control methods that target these genes can be explored. We conducted an RNA-seq experiment that revealed 2479 differentially expressed genes between pheromone-producing and non-pheromone-producing boll weevils. Of those genes, 1234 were up-regulated, and 1515 were down-regulated, and most had gene annotations associated with pheromone production, development, or immunity. This work advances our understanding of boll weevil pheromone production and brings us one step closer to developing gene-level control strategies for this cotton pest.

## 1. Introduction

The boll weevil, *Anthonomus grandis grandis* Boheman (Coleoptera: Curculionidae), is a pest of commercial cotton (*Gossypium hirsutum*) throughout the Americas [1]. Efforts to eradicate the boll weevil in the United States were initiated in the late 1970s, and since that time, the boll weevil has been eliminated from most of its U.S. range [2,3,4]. However, boll weevil populations remaining in the Lower Rio Grande Valley (LRGV) production area of Texas continue to pose a threat to other cotton-growing areas in the state and neighboring states (https://www.txbollweevil.org.html accessed on 19 September 2021). In fact, recent infestations in eradicated areas in Texas have resulted in multi-year infestations, which have cost millions of dollars to remediate (P. Burson, personal communication).

Eradication programs rely exclusively on pheromone traps to detect and monitor boll weevil populations [5]. Pheromone traps are typically placed next to cotton fields and monitored weekly during the active phase of eradication. Each trap is baited with a pheromone lure containing a nominal dose of 10 mg of grandlure (synthesized boll weevil pheromone), which is composed of four pheromone components: two terpene alcohols (components I and II; (+)-cis-2-isopropenyl-1-methyl cyclobutaneethanol and cis-3,3-dimethyl-Δ ^1,β^-cyclohexaneethanol, respectively) and two terpene aldehydes (components III and IV; cis-3,3-dimethyl- Δ ^1,α^-cyclohexaneacetaldehyde and trans-3,3-dimethyl- Δ ^1,α^-cyclohexaneacetaldehyde) [6]. The pheromone is produced by male weevils and is attractive to both sexes and subsequently considered an aggregation rather than a sex pheromone [7]. During post-eradication, programs may place traps in close proximity to cotton fields or in a grid-like manner irrespective of the location of cotton fields. Traps are monitored every two or three weeks, and use lures dosed with 25 mg of grandlure and 30 mg of eugenol. Regardless, both active and post-eradication programs rely on traps to indicate the presence of weevils and the need for remedial actions.

It is generally recognized that weevil captures in traps vary seasonally and decline rapidly once cotton begins to produce fruiting structures [8,9,10]. In fact, some cotton fields may be heavily infested with boll weevils, yet adjacent traps may not capture any weevils [11]. Such scenarios could result in fields serving as nurseries for boll weevils, producing thousands to millions of boll weevils throughout the growing season if left untreated. Several reasons for the reduced performance of pheromone traps during the fruiting stage of cotton development have been proposed [8,9,10,12]. The most widely accepted reason is that weevils producing pheromone in the field outcompete the pheromone released from traps [13,14,15,16,17]. Nevertheless, once a weevil is detected in a trap, a malathion application is triggered for the respective field or growing area. Malathion treatments may continue weekly until weevils are no longer detected. Consequently, a field may receive as many as 25 applications in a single growing season (P. Burson, personal communication). Thus, new control methods are needed that are cleaner and safer for humans and the environment surrounding cotton fields and, ideally, specific to the boll weevil.

One way to avoid trap competition in the field is to develop a gene-based control strategy that suppresses the production of pheromone in the male weevil. This could increase the attractiveness of the pheromone lure in traps allowing for more reliable and early detection of weevils; thus, leading to more timely insecticide applications. Suppression of pheromone-producing weevils could also reduce and/or delay early-season colonization of cotton fields by overwintered weevils. Such delays or reductions could potentially prevent fields from becoming heavily infested [18], which in turn could reduce malathion applications per season. Here we present a boll weevil transcriptome created using next-generation RNA sequencing with a BUSCO score of 99.27%. We used the transcriptome to conduct an RNA-seq experiment to identify genes involved in pheromone production and pheromone biosynthesis of male boll weevils.

## 2. Materials and Methods

### 2.1. Boll Weevil Collections and Experimental Design

Boll weevils were initially collected as larvae in infested squares from a field northwest of Edinburg, TX (26.5°N, 98.2°W). The major life stages (early instar larvae, late instar larvae, pupae, adult male, and adult female) were placed in RNAlater (Invitrogen, Carlsbad, CA, USA), and stored at −20 °C until total RNA was extracted for the transcriptome assembly. A group of pupae from the same field was kept on vermiculite in a 29.4 ± 1 °C degree incubator to obtain adults for the pheromone experiment. Newly eclosed adults (<24 h old) were sexed, and only the males were kept. Forty males were divided into two groups. One group was held without food for 24 h. The other group was fed squares (6–9 mm in diameter; bracts intact) daily for 6–8 days and provided water on a section of cotton dental wick saturated with deionized water to promote the production of all four pheromone components [19,20]. Weevils in both groups were tested for pheromone production using gas chromatography-mass spectrometry and procedures described by Suh and Spurgeon (2016) [21]. Ten weevils in each group (i.e., pheromone-producing or non-pheromone-producing) were placed in 2-mL snap cap tubes with RNAlater and stored at −20 °C until nucleic acid was extracted.

### 2.2. RNA Extraction and Sequencing

RNA was extracted from weevils in each life stage using the RNeasy extraction kit (Qiagen, Hilden, Germany). Samples were checked for quantity and quality using a Tapestation 4200 (Agilent, Santa Clara, CA, USA). The total RNA from 12 samples at different life stages (two second instar larvae; two third instar larvae; two pupae; two adult females; and two adult males) and 20 weevils from the pheromone experiment (10 pheromone-producing males and 10 non-pheromone-producing males) were submitted to Texas A&M AgriLife Genomics and Bioinformatics Service (TxGen) for purification, library preparation, and sequencing on an Illumina NovaSeq (Illumina, San Diego, CA, USA), paired-end with a read length of 2 × 150 base pairs.

### 2.3. Transcriptome Assembly and Annotation

A total of approximately 30 million reads per sample were downloaded from TxGen and trimmed of adaptors and low-quality sequences using Trimmomatic 0.38 [22]. Approximately 97% of all life stage reads passed quality control measures resulting in a total of 352,189,343 sequences. Raw sequences are available at NCBI SRA (PRJNA734329). The trimmed reads were used to assemble a *de novo* transcriptome using the TRINITY 2.12.0 assembler [23]. The default parameters were used, including the options for pair-wise reads, non-strand specific assembly, minimum codon length of 200 bp, and the construction of super contigs. CD-HIT was used to cluster the sequences and reduce the number of isomers [24]. Completeness of the transcriptome was assessed using Benchmarking Universal Single-Copy Orthologs (BUSCO) [25,26,27]; selected from OrthoDB using the insecta database [28]. The transcriptome was annotated using CloudBLAST and InterProScan via OmicsBox (Valencia, Spain). Parameters were limited to the insecta database and E-value ≤ e × 10^−5^.

### 2.4. RNA-Seq Analysis

A total of approximately 30 million reads per sample (586,574,829 total reads) for the pheromone and non-pheromone samples were used in the RNA-seq analysis, which was accomplished using edgeR as part of the OmicsBox software [29]. Because no reference genome was available for pair-wise differential expression analysis, the reads were mapped to the *de novo* transcriptome using RNA-seq by Expectation-Maximization (RSEM). The workflow also included quantifying the mapped reads at each locus and statistically assessing fold change (False Discovery Rate (FDR) < 0.05) differences between pheromone-producing and non-pheromone-producing weevils [30]. Individual genes with relevant Gene Ontology (GO) terms were further examined manually. The complete list of differentially expressed genes (DEGs) can be found in Supplemental Table S1.

## 3. Results and Discussion

### 3.1. Transcriptome

The final compiled transcriptome had 195,128 total transcripts with an average length of 922.89 bp, Table 1. We assessed the completeness of the transcriptome by comparing our assembled transcripts to conserved orthologs found in other insects using BUSCO [25,26,27]. The transcriptome had a BUSCO score of 99.27% with only 0.59% and 0.15% fragmented or missing, respectively, Table 1.

**Figure 1:**
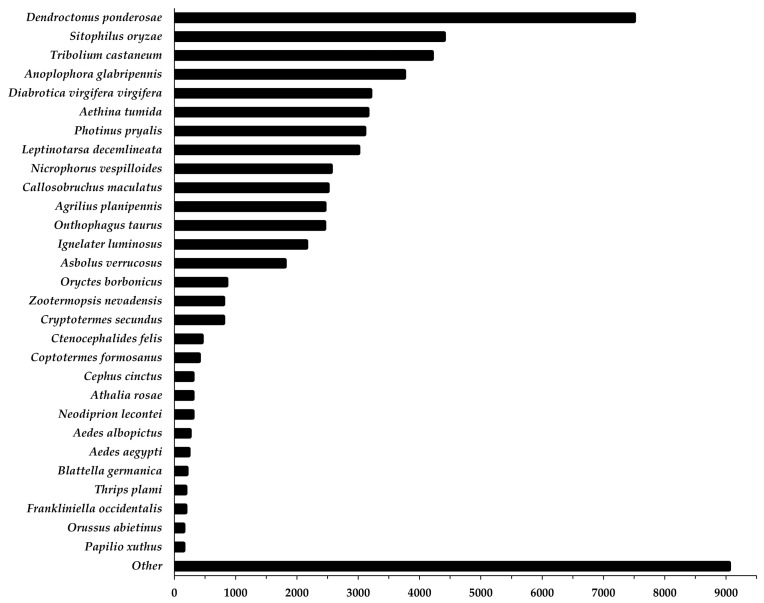
BLAST top species hits for the annotated *Anthonomus grandis grandis de novo* transcriptome. Top hit species are listed on the *y*-axis, and the number of hits for each species is on the *x*-axis.

The BLAST annotation revealed most sequences aligned to two weevil species, *Dendroctonus ponderosae* Hopkins and *Sitophilus oryzae* Linnaeus, followed by the tenebrionid, *Tribolium castaneum* Herbst. Nearly all sequences in the dataset aligned to other coleopterans, Figure 1.

**Figure 2:**
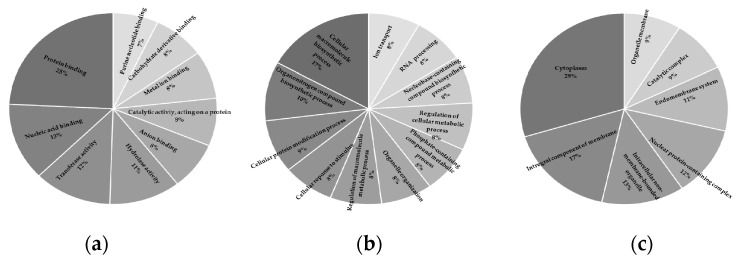
Gene ontology categories for the annotated *Anthonomus grandis grandis de novo* transcriptome indicating (**a**) molecular function (MF), (**b**) biological process (BP), and (**c**) cellular component (CC).

Gene Ontology (GO) was performed for all three major categories: molecular function (MF), biological process (BP), and cellular component (CC). Standard gene groups were represented in all three categories, Figure 2. The largest categories for MF were protein binding (24%) and nucleic acid binding (13%), Figure 2a. Top ontologies for BP were cellular macromolecule biosynthetic process (17%) and organonitrogen compound biosynthetic process (10%), Figure 2b. CC GO terms were the highest for cytoplasm (30%) and an integral component of membrane (17%), Figure 2c.

### 3.2. RNA-Seq Analysis

**Figure 3:**
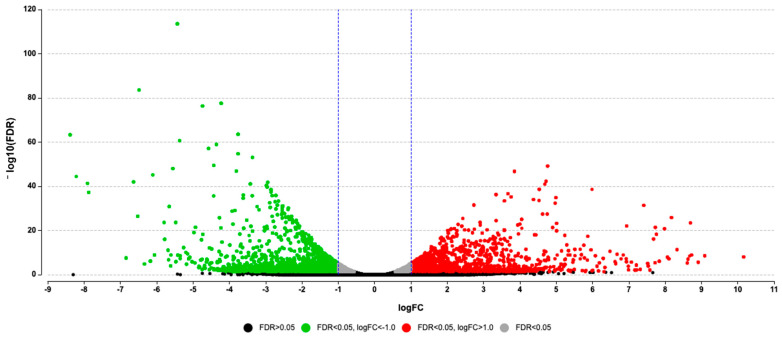
Volcano Plot showing the 2479 significant differentially expressed genes between *Anthonomus grandis grandis* males that produced pheromone and those that did not produce pheromone. Of these genes, 1234 were up-regulated (red; FDR < 0.05, logFC > 0.01) and 1515 genes were down-regulated (green; FDR < 0.05, logFC < 0.01). Grey dots represent genes with FDR < 0.01, and black dots represent the majority of genes with FDR > 0.05. The blue vertical lines show the −1 and 1 log fold change threshold. The *y*-axis shows the negative log10 FDR value, and the *x*-axis shows the log2 fold change in gene expression.

RNA-seq analysis identified a total of 2479 DEGs between the males that produced pheromone compared to those that did not produce pheromone. Of those 2479 genes, 1234 genes were up-regulated, and 1515 genes were down-regulated, Figure 3 and Supplemental Table S1.

**Figure 4:**
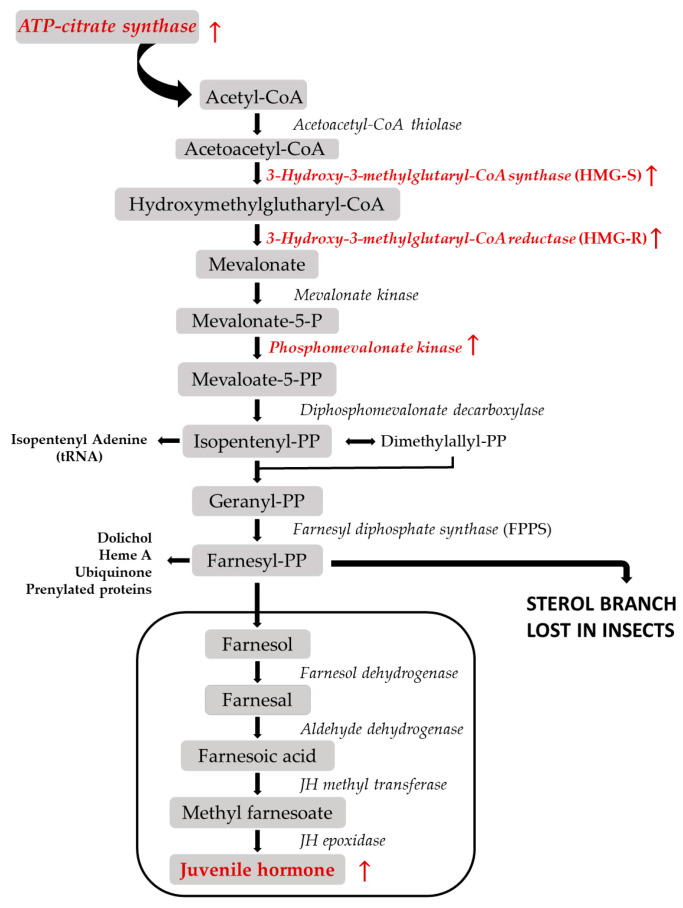
Mevalonate pathway in insects. Genes that were up-regulated in pheromone-producing *Anthonomus grandis grandis* are highlighted in red and accompanied by an up-facing arrow. This figure was modified from Bellès et al., 2005 [32].

Evidence from other coleopterans shows pheromone is made by utilizing the first section of the juvenile hormone pathway, called the mevalonate pathway, which uses byproducts to produce the monoterpenoid pheromone component [31]. The remaining steps in the juvenile hormone pathway produce juvenile hormones, which are critical in the regulation of molting, development, and induction of pheromone production. Thus, pheromone production and insect development are correlated phenotypes relying, at least partially, on the same biochemical pathway. Additionally, we established the pheromone-producing and non-pheromone-producing phenotypes by (1) withholding food from newly eclosed weevils and (2) allowing weevils that were given food 6–8 days to stimulate the production of pheromone [20]. Thus, differences in the age and nutrient availability between treatment groups also likely contributed to the differences observed between pheromone-producing and non-pheromone-producing boll weevils. With that in mind, we first discuss genes that have a direct function in pheromone production and pheromone biosynthesis that were significantly DE between treatment groups, Table 2 and Figure 4. We then discuss other interesting genes found within the top 50 significantly up- and down-regulated DEG genes (based on FDR value) and make predictions on their function based on GO term descriptions and the literature, Table 2 and Table 3.

The mevalonate pathway in insects is a metabolic pathway initiated by reductive polymerization of acetyl-CoA and leads to a diversity of isoprenoid compounds [32]. Information on pheromone production and the mevalonate pathway in insects has been studied mostly in pine beetles and Drosophila; however, more studies working to elucidate the steps in the pathway are becoming available [31,33]. In this study, we detected eight genes directly involved in the mevalonate pathway that were up-regulated in pheromone-producing males, Table 2 and Figure 4. For example, *hydroxymethylglutaryl-CoA synthase 1 (HMG-S), 3-hydroxy-3-methylglutaryl-coenzyme* A *reductase (HMG-R)*, and *phosphomevalonate kinase* were all highly up-regulated and found in the first steps of the mevalonate pathway converting acetyl-CoA to mevalonate 5-PP, the final step before isopentenyl synthesis, Figure 4. Additionally, our study identified *ATP-citrate synthase*, which converts citrate to acetyl-CoA, and is needed to initiate the mevalonate pathway, Table 2 and Figure 4.

Our study found up-regulation in genes in the isopentenyl, dolicol, and farnesyl portions of the pathway as well, Table 2 and Figure 4. We also found a gene involved in the processes that transfers sugar to glycoproteins during the dolichol pathway, *dolichyl pyrophosphate*. Similar molecules have also been shown to be part of the production of isoprenoid compounds in blowfly larvae [34]. Several up-regulated genes had links to the accumulation of lipids or lipid metabolism, *fatty acid synthase*, and *glycerol-3-phosphate phosphatase*, and most likely function to increase the size of the fat body and hormone production. The increase in a particular type of fat body is a feature that correlates with reproductive maturity in the boll weevil [20]. Furthermore, Tittiger and Blomquist (2016) reported that pine bark beetle pheromone also consists of components derived from fatty acids, which could also be the case in the boll weevil [35]. There were seven juvenile hormones (JH) up-regulated in pheromone-producing weevils. The final steps of the mevalonate pathway result in the production of JHs. While JHs have many functions in insects, they have been shown to regulate isoprenoid pheromone production in the midgut of *I. pini* males and are speculated to regulate the mevalonate pathway [36].

We also identified two genes that may function to stimulate hormone production in pheromone-producing male boll weevils, *shuttle craft-like* and *regucalcin-like*, Table 2. The gene *shuttle craft* has mostly been studied in its role in the embryonic central nervous system development in *D. melanogaster* [37], for which it is required. It also has been shown to have an expression in the nervous system and reproductive system in adult male and female flies. It has been speculated that *shuttle craft* regulates genes that are either involved in axon growth or the secretion of molecules needed for chemoattraction [38]. Interestingly, it was up-regulated in pheromone-producing male boll weevils and may function in laying down the neurons needed to produce pheromones. Likewise, the protein *regucalcin-like* has been studied very little in insects but has been characterized in mammals as a calcium-binding protein that regulates many cell functions needed for hormonal stimulation [39]. Thus, both genes seem to be involved in hormone stimulation that may be needed to activate the pheromone production pathway in the boll weevil.

Aside from genes directly involved in pheromone production, we also found genes up-regulated in associated processes, such as digestion and immunity. The most highly up-regulated gene in the dataset was a serine protease, transmembrane protease serine 9-like, Table 3. In fact, there were six serine protease genes up-regulated in the 50 most highly up-regulated gene list, Supplemental Table S1. Serine proteases serve many cellular functions, including development, apoptosis, and digestion [40,41]. The most highly expressed serine peptidase in this study had a high sequence identity to a gut serine peptidase in *T. castaneum* that was highly induced in response to feeding [42]. Thus, it is likely that *transmembrane protease serine 9-like* is also involved in digestion in the boll weevil. We found six other DE serine peptidase sequences, outside of the top 50 gene list, that had *A. grandis* as a top BLAST hit and were previously identified as midgut serine proteases [43]. In that study, two of these genes, *Agser2p* and *Agser5p*, were induced in response to feeding. The up-regulation of digestive peptidase genes in our dataset is fitting because pheromone-producing males were fed fresh squares daily whereas, the non-pheromone-producing males were not given food. The remaining up-regulated serine peptidase genes could be involved in digestion as well but may also have roles in development and apoptosis, both of which may be necessary for boll weevil sexual maturity and pheromone production.

Two immune genes were differentially expressed protein spaetzle 3 and DNA/RNA non-specific nuclease 1. In Drosophila, spaetzle is involved in embryonic development but also has a role in the innate immune response. Spaetzle proteins bind to Toll receptors and activate antimicrobial peptide gene expression [44]. The second gene, *DNA/RNA non-specific nuclease 1*, was shown to be in the midgut of *Bombyx mori* and interferes with the RNA interference response. Because of its propensity to degrade dsRNA, it has been suggested the enzyme might be involved in the innate immune response against invading nucleic acids, such as RNA viruses [45]. The functionality and increased expression in innate immune genes in pheromone-producing weevils is not well understood, but some studies suggest that male pheromone quality is a direct assessment of overall male health [46]. In fact, a study using *Tenebrio molitor*, showed males were more attractive to females if they were immunocompetent [47]. Thus, having a good immune system may affect the overall quality of pheromones.

Many of the genes that were significantly down-regulated in pheromone-producing weevils had functions consistent with the cuticle and some were directly involved in cuticle tanning and sclerotization, Table 3. This is the process where the cuticle darkens (melanin production), and the exoskeleton becomes hard [48,49]. This process is achieved through the tyrosine metabolic pathway and has been characterized mainly in the model beetle, *T. castaneum*. The gene *laccase 2* was found to play a major role in cuticle tanning for all life stages except the egg stage [50]. Likewise, this gene was decreased nearly 19-fold in this study, Table 3. Dopamine also plays a critical role in the conjugation of β-alanine to produce Ν-β -alanyldopamine, which directly leads to pigmentation precursors [51]. We found two dopamine-related genes in our dataset, *dopamine D2-like receptor* and *dopamine N-acetyltransferase*, that may play a part in weevil cuticle tanning. Additionally, we found four cuticle genes, two chitin synthase genes, and many other cuticle genes outside of the top 50 DEGs, all down-regulated in pheromone-producing weevils, Table 3 and Supplemental Table S1. This pathway is most likely highlighted in our data because the pheromone-producing weevils were 6 to 8 days older compared to the non-pheromone-producing weevils. Thus, pheromone-producing weevils were likely finished with the tanning process, while non-pheromone-producing males, which were less than 24 h old, still had soft cuticles that were not fully sclerotized and had not become dark.

The gene *transcription factor ken*, was also significantly down-regulated in pheromone-producing boll weevils, Table 3. Interestingly, the ortholog in *D. melanogaster*, a transcription factor named *ken* and *barbie*, has a distinct role in the development of male and female external genitalia and is expressed in the highest amounts during embryo and pupal stages of development. In fact, mutants of this gene gave a range of deformities, including no external genitalia [52]. This is another example of an interesting gene to target with gene-based pest control strategies because reduced expression at key periods of insect development could lead to males with completely absent external reproductive organs, thus reducing successful mating in the field.

## 4. Conclusions

We provide a boll weevil *de novo* transcriptome with a BUSCO score of 99.27%. We used this assembly to perform an RNA-seq analysis comparing gene expression between pheromone-producing and non-pheromone-producing boll weevils. We ultimately identified significant DEGs that likely have key functions in pheromone production and biosynthesis. In subsequent experiments, successful knockdown of these DEGs should result in boll weevils with no or limited pheromone production, which could result in increased detection of weevils with traps. Because eradication programs in the U.S. rely almost exclusively on pheromone traps to indicate the need for insecticide applications, improved detection of boll weevils with traps could lead to more timely application of insecticides. Furthermore, disruption of boll weevil pheromone production may reduce early-season colonization of weevils that rely on pheromone to find cotton. Finally, the other DEGs identified in this study involved in immunity, cuticle formation, and digestion may also serve as targets for gene-level pest control. Thus, this RNA-seq analysis provides the foundation for the production of gene-level control strategies that may target pheromone production and other life functions in the boll weevil. Control strategies that incorporate these methods, such as RNA interference or CRISPR, would not only be specific to the boll weevil but would also be safe and environmentally friendly.

## Figures and Tables

**Table 1:** *Anthonomus grandis grandis* de novo transcriptome statistics and BUSCO scores.

Raw sequences	352,189,343
Total transcripts	195,128
Average sequence length	922.89 bp
N50	2040 bp
BUSCO	
Complete	99.27%
Complete single copy	97.81%
Complete duplicated	1.46%
Fragmented	0.59%
Missing	0.15%

Underline shows that the information below describes the BUSCO score.

**Table 2:** List of genes consistent with pheromone production and biosynthesis that were up-regulated in pheromone-producing *Anthonomus grandis grandis* compared to non-pheromone-producing boll weevils.

Sequence Name	Sequence Description	BLAST Top Hit	Fold Change	UP/DOWN	*p*-Value	FDR
Mevalonate pathway or associated with pheromone production and biosynthesis						
TRINITY_DN11108	hydroxymethylglutaryl-CoA synthase 1 (HMG-S)	*D. ponderosae*	23.08	↑	1.45 × 10^−3^^7^	2.67 × 10^−3^^4^
TRINITY_DN13668	dolichyl pyrophosphate Man9GlcNAc2 α-1,3-glucosyltransferase	*D. ponderosae*	2.01	↑	4.44 × 10^−0^^5^	1.82 × 10^−0^^3^
TRINITY_DN5050	farnesyl pyrophosphate synthase-like	*I. pini*	11.77	↑	1.30 × 10^−0^^4^	4.59 × 10^−0^^3^
TRINITY_DN5422	phosphomevalonate kinase	*D. ponderosae*	3.05	↑	9.80 × 10^−1^^2^	1.51 × 10^−0^^9^
TRINITY_DN8104	farnesyl pyrophosphate synthase	*I. pini*	34.05	↑	1.10 × 10^−0^^5^	5.33 × 10^−0^^4^
TRINITY_DN8499	isopentenyl-diphosphate Delta-isomerase 1	*A. glabripennis*	37.64	↑	1.57 × 10^−0^^9^	1.69 × 10^−0^^7^
TRINITY_DN873	3-hydroxy-3-methylglutaryl-coenzyme A reductase-like (HMG-R)	*I. paraconfusus*	16.57	↑	9.18 × 10^−2^^9^	9.18 × 10^−2^^6^
TRINITY_DN4352	ATP-citrate synthase	*D. ponderosae*	6.66	↑	1.89 × 10^−2^^5^	3.07 × 10^−3^^2^
Juvenile hormone						
TRINITY_DN11992	juvenile hormone inducible protein	*D. ponderosae*	7.75	↑	7.40 × 10^−1^^3^	1.39 × 10^−1^^0^
TRINITY_DN4315	juvenile hormone esterase-like	*D. ponderosae*	2.98	↑	5.14 × 10^−1^^1^	7.24 × 10^−0^^9^
TRINITY_DN874	juvenile hormone esterase-like	*D. ponderosae*	2.71	↑	1.99 × 10^−0^^8^	1.79 × 10^−0^^6^
TRINITY_DN4184	juvenile hormone binding protein	*R. ferrugineus*	5.64	↑	2.69 × 10^−0^^8^	2.34 × 10^−0^^6^
TRINITY_DN9052	juvenile hormone inducible protein	*D. ponderosae*	4.53	↑	7.72 × 10^−0^^7^	4.94 × 10^−0^^5^
TRINITY_DN115965	putative juvenile hormone inducible protein	*D. ponderosae*	3.26	↑	1.10 × 10^−0^^5^	5.33 × 10^−0^^4^
TRINITY_DN11903	juvenile hormone acid O-methyltransferase	*D. ponderosae*	2.89	↑	5.87 × 10^−0^^5^	2.31 × 10^−0^^3^
Fatty acid metabolism						
TRINITY_DN58	fatty acid synthase	*D. ponderosae*	4.92	↑	2.20 × 10^−2^^6^	1.74 × 10^−2^^3^
TRINITY_DN5986	glycerol-3-phosphate phosphatase-like	*S. oryzae*	7.50	↑	3.68 × 10^−2^^6^	2.89 × 10^−2^^3^
Neurological and hormonal regulation						
TRINITY_DN4411	protein shuttle craft like	*D. ponderosae*	5.38	↑	3.60 × 10^−2^^9^	3.64 × 10^−2^^6^
TRINITY_DN72610	regucalcin-like	*D. ponderosae*	5.28	↑	1.45 × 10^−2^^5^	1.11 × 10^−2^^2^

**Table 3:** List of top up- and down-regulated genes in pheromone-producing *Anthonomus grandis grandis* compared to non-pheromone-producing boll weevils. Rows highlighted in light grey represent genes that were DE but not part of the top 50 DEGs.

Sequence Name	Sequence Description	Blast Top Hit	Fold Change	UP/DOWN	*p*-Value	FDR
Peptidase activity						
TRINITY_DN62502	transmembrane protease serine 9-like	*Z. cucurbitae*	27.18	↑	1.17 × 10^−53^	7.28 × 10^−50^
TRINITY_DN35065	cathepsin L1-like	*R. ferrugineus*	14.42	↑	3.42 × 10^−51^	1.73 × 10^−47^
TRINITY_DN2409	carboxypeptidase B-like	*D. ponderosae*	12.74	↑	9.29 × 10^−^^41^	2.28 × 10^−^^37^
TRINITY_DN5051	venom serine carboxypeptidase-like	*S. oryzae*	26.91	↑	2.87 × 10^−31^	3.46 × 10^−28^
TRINITY_DN13263	trypsin-like serine prtoease	*A. grandis*	15.38	↑	4.09 × 10^−26^	3.18 × 10^−23^
TRINITY_DN4893	venom serine protease-like	*D. ponderosae*	10.49	↑	2.70 × 10^−22^	1.45 × 10^−19^
TRINITY_DN34699	trypsin alpha 3-like (Agser2p)—induced by eating	*A. grandis*	3.83	↑	6.99 × 10^−15^	1.76 × 10^−12^
TRINITY_DN35978	brachyurin-like (Agser9p)	*A. grandis*	3.36	↑	9.32 × 10^−12^	1.46 × 10^−09^
TRINITY_DN4825	brachyurin-like (Agser5p)—induced by eating	*A. grandis*	2.26	↑	5.60 × 10^−06^	2.93 × 10^−04^
TRINITY_DN49721	brachyurin-like (Agser9p)	*A. grandis*	3.56	↑	9.43 × 10^−13^	1.74 × 10^−10^
TRINITY_DN63273	trypsin alpha-3-like (Agser2p)—induced by feeding	*A. grandis*	3.57	↑	2.63 × 10^−13^	5.21 × 10^−11^
TRINITY_DN69718	serine protease (Agser12p)	*A. grandis*	2.27	↑	2.86 × 10^−05^	1.24 × 10^−03^
Immune response						
TRINITY_DN4819	protein spaetzle 3	*D. ponderosae*	25.78	↑	3.30 × 10^−45^	1.11 × 10^−41^
TRINITY_DN3567	DNA/RNA non-specific nuclease 1	*A. grandis*	13.54	↑	3.13 × 10^−39^	6.34 × 10^−36^
Sexual maturity						
TRINITY_DN10824	transcription factor Ken		7.86	↓	1.35 × 10^−44^	4.39 × 10^−41^
Cuticle tanning and sclerotization						
TRINITY_DN16013	pupal cuticle protein 36-like	*D. ponderosae*	89.53	↓	6.46 × 10^−89^	2.61 × 10^−84^
TRINITY_DN11117	cuticular protein 141	*D. ponderosae*	13.54	↓	1.68 × 10^−68^	2.72 × 10^−64^
TRINITY_DN3947	chitin deacetylase 4	*D. ponderosae*	20.48	↓	1.14 × 10^−63^	1.16 × 10^−59^
TRINITY_DN4706	chitin synthase 1	*A. grandis*	5.84	↓	5.48 × 10^−35^	8.37 × 10^−32^
TRINITY_DN1999	cuticle protein 19.8-like	*S. oryzae*	15.15	↓	1.49 × 10^−32^	1.89 × 10^−29^
TRINITY_DN4303	cuticular protein analogous to peritrophins	*D. ponderosae*	5.84	↓	1.53 × 10^−32^	1.91 × 10^−29^
TRINITY_DN2312	laccase 2	*A. eugenii*	18.71	↓	9.87 × 10^−83^	2.66 × 10^−78^
TRINITY_DN22343	dopamine D2-like receptor	*S. oryzae*	4.85	↓	4.95 × 10^−09^	4.90 × 10^−07^
TRINITY_DN6738	dopamine N-acetyltransferase	*D. ponderosae*	2.03	↓	4.40 × 10^−04^	1.30 × 10^−02^

## Data Availability

The submission of raw sequences to NCBI SRA has been started under project number PRJNA734329.

## Supplementary Materials

The following are available online at https://www.mdpi.com/article/10.3390/insects12100893/s1, Table S1: Complete list of 192 differentially expressed genes between pheromone-producing and non-producing boll weevils.
